# An immune-competent human gut microphysiological system enables inflammation-modulation of *Faecalibacterium prausnitzii*

**DOI:** 10.21203/rs.3.rs-3373576/v1

**Published:** 2023-10-12

**Authors:** Jianbo Zhang, Yu-Ja Huang, Martin Trapecar, Charles Wright, Kirsten Schneider, John Kemmit, Victor Hernandez-Gordillo, Jun Young Yoon, Eric J. Alm, David T. Breault, David Trumper, Linda G. Griffith

**Affiliations:** 1 Department of Biological Engineering, Massachusetts Institute of Technology, Cambridge, MA, USA; 2 Department of Pediatrics, Harvard Medical School, Boston, MA, USA; 3 Department of Mechanical Engineering, Massachusetts Institute of Technology, Cambridge, MA, USA; 4 Center for Gynepathology Research, Massachusetts Institute of Technology, Cambridge, MA, USA; 5 Swammerdam Institute for Life Sciences, University of Amsterdam, Amsterdam, The Netherlands; 6 Department of Mechanical Engineering, Yonsei University, Seoul, South Korea

**Keywords:** Gut microbiome, organ-on-a-chip, host-microbiome crosstalk, *Faecalibacterium duncaniae*, GuMI

## Abstract

Crosstalk of microbes with human gut epithelia and immune cells is crucial for gut health. However, there is no existing system for a long-term co-culture of human innate immune cells with epithelium and oxygen-intolerant commensal microbes, hindering the understanding of microbe-immune interactions in a controlled manner. Here, we establish a gut epithelium-microbe-immune microphysiological system to maintain the long-term continuous co-culture of *Faecalibacterium prausnitzii/Faecalibacterium duncaniae* with colonic epithelium, antigen-presenting cells (APCs, herein dendritic cells and macrophages), with CD4^+^ naïve T cells circulating underneath the colonic epithelium. Multiplex cytokine assays suggested that APCs contribute to the elevated level of cytokines and chemokines being secreted into both apical and basolateral compartments. In contrast, the absence of APCs does not allow reliable detection of these cytokines. In the presence of APCs, *F. prausnitzii* increased the transcription of pro-inflammatory genes such as toll-like receptor 1 (TLR1) and interferon alpha 1 (IFNA1) in the colonic epithelium, but no significant change on the secreted cytokines. In contrast, integration of CD4^+^ naïve T cells reverses this effect by decreasing the transcription of TLR1, IFNA1, and indoleamine 2,3-dioxygenase, and increasing the *F. prausnitzii*-induced secretion of pro-inflammatory cytokines such as IL-8, MCP-1/CCL2, and IL1A. These results highlight the contribution of individual innate immune cells in the regulation of the immune response triggered by the gut commensal *F. prausnitzii*. The successful integration of defined populations of immune cells in this gut microphysiological system demonstrated the usefulness of the GuMI physiomimetic platform to study microbe-epithelial-immune interactions in health and disease.

## Introduction

The human colonic mucosal barrier is a microarchitecture that acts as a physical barrier to harmful pathogens and as a coordinator of homeostatic crosstalk between microbiota and immune cells.^1^ Distinct from the small intestine, the colon does not have big villi, resulting in a relatively flat epithelial surface composed of a single layer of cells (i.e., monolayer).^1,2^ This colonic epithelial monolayer consists of several cell types, including colonocytes, goblet cells, Tuft cells, and endocrine cells.^1^ These cells communicate with microbiota by actively metabolizing microbial metabolites (e.g., butyrate) and secreting host molecules (e.g., mucin). In addition, the epithelial cells communicate with innate immune cells, which release cytokines/chemokines or secrete IgA to prevent the body from bacterial invasion.^3,4^ Antigen-presenting cells (APCs), including dendritic cells and macrophages, are essential for immune tolerance and protective immunity in the intestine. These cells perform distinct functions and are differentially modulated by the microbiota to perform these roles.^5^ Disruption of this microbiota-epithelium-immune axis can lead to inappropriate immune responses, which is believed to contribute to the development or progression of inflammatory diseases, including inflammatory bowel diseases.^6^ However, the precise role of each component in the microbiota-epithelium-immune axis remains elusive, mainly owing to the lack of an appropriate model to disentangle the complex interactions.

While there is progress being made in developing humanized *in vitro* microfluidic gut models to mimic the microbiota-immune interplay, current *in vitro* microfluidic systems often use cancerous cell lines,^7^ lack immune components^8^ or use PBMCs^9^ comprising an undefined mixture of immune cells. In addition, these microfluidic gut chips are fabricated from polydimethylsiloxane (PDMS),^8,9^ a material that is highly adsorptive of lipophilic molecules such as secreted cytokines or chemokines, which are critical for the innate immune responses. The discrepant oxygen requirement further expands these challenges: the majority of >1000 bacterial species in the colon are intolerant to oxygen, whereas human intestinal and immune cells require oxygen. We recently established a primary human cell-derived non-PDMS gut-liver physio-mimetic system and demonstrated a reliable co-culture of differentiated colonic epithelium, APCs, and regulatory T cells for studying inflammatory bowel disease^10^ and neurodegenerative diseases.^11^ A new gut-microbe (GuMI) microphysiological system was developed in parallel. GuMI enabled the continuous culture of colonic epithelium with the oxygen-sensitive bacterium *Faecalibacterium prausnitzii*, which constitute the majority of the colonic microbiota^12^ and has important implication in reducing the risk of inflammatory diseases.^13^

Here, we describe the establishment of an immune-competent GuMI platform for co-culturing three types of immune cells (dendritic cells, macrophages, and CD4^+^ naïve T cells) with a primary human colonic epithelium and *F. prausnitzii* over 48 hours. We examined the influence of human monocyte-derived APCs, aka dendritic cells and macrophages, on the phenotype of the colon mucosal barrier and the bacterial growth compared to GuMI without immune cells, assessing barrier function, bacterial growth, and cytokine profile. We then studied the effects of *F. prausnitzii* in a multi-day interaction with primary human colonic epithelium, APCs, and CD4^+^ naïve T cells. By measuring the gene transcription and cytokine secretion, we found the specific effects of *F. prausnitzii* on APC-mediated immune responses. In addition, including CD4+ naïve T cells in the system reduces transcription of pro-inflammatory genes in the epithelium but increases cytokine secretion to the luminal side of the colonic epithelium.

## Results and Discussion

### Antigen-presenting cells do not disrupt the integrity of colonic epithelium nor the growth of F. prausnitzii

Epithelial mucosal barriers regulate their homeostasis and response to microbiota in part by collaboration with innate immune cells and polarized production of growth factors, chemokines, and cytokines.^14^ Most of these factors act not only in a paracrine fashion to recruit immune cells and signal neighboring stromal cells but also in an autocrine fashion: colonic epithelial cells and innate immune cells express not only canonical growth factor receptors (e.g., epidermal growth factor receptor, EGFR; fibroblast growth factor receptors, FGFRs; platelet-derived growth factor receptors, PDGFRs) but also receptors for chemokines (CXCR1-4; CCR2-5) and cytokines (receptors IL-1, IL-4, IL-15, and IL-18).^15–23^ Autocrine loops regulate colonic epithelial barrier permeability, proliferation, response to infection, and diverse other behaviors, and in turn, the activity of autocrine loops is influenced by gut microbes.^14^

**Figure 1.**
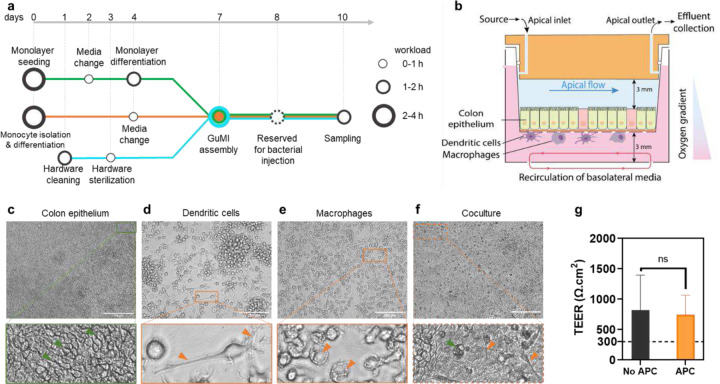
Establishment and characterization of the co-culture of colonic epithelium, dendritic cells, and macrophages in the GuMI system. (**a**) workflow of colonic epithelial monolayer generation (green line), monocyte isolation, and differentiation to antigen-presenting cells (APCs, i.e., dendritic cells and macrophages; orange line), GuMI hardware preparation (aqua line), GuMI device assembly, operation, and sampling (merged lines). Circles in the metro map indicate the critical tasks and the workload. (**b**) illustration of designed co-culture of primary colonic epithelium with APCs in GuMI (GuMI-APC). (**c-f**) brightfield images of colonic epithelial monolayer, dendritic cells, macrophages, and co-culture. (**c**) colonic epithelium without APCs in GuMI. Green arrows indicate the clear cell border among the epithelial cells. (**d**) dendritic cells and (**e**) macrophages before adhering to the bottom of the semi-permeable membrane in the transwell insert. Orange arrows indicate the dendrite and phagosome-like structures in (d) and (e). Scale bars in (**c-f**): 300 µm. (**g**) Transepithelial electrical resistance (TEER) values of the monolayer in GuMI-APC (orange bar) and GuMI (black bar) after 72 h in GuMI. The error bar indicates the standard deviation. N=3.

We previously established a GuMI system to co-culture colonic epithelium and anaerobic bacteria. However, the system lacked immune components, hampering the study of crosstalk among epithelial cells, immune cells, and bacteria. Reasoning that cytokine and chemokine secretion are essential markers and regulators of the immune responses to the gut microbiota and the lack of immune cells in the GuMI system leads to a negligible level of cytokines and chemokines (data not shown), we integrated innate immune cells into the GuMI system. We isolated the monocytes from human primary peripheral blood mononuclear cells (PBMCs) and deliberately differentiated the monocytes into two types of antigen-presenting cells (APCs), namely dendritic cells and macrophages (more details in Methods, Figure 1a). These dendritic cells and macrophages were attached on the basolateral side of the collagen-coated membrane that supports the colonic epithelial monolayers before they were integrated into the GuMI platform under a physiological oxygen gradient where the apical compartment was maintained anaerobic (Figure 1b). Colonic epithelium and APCs were co-cultured in the sandwich-like co-culture to allow the epithelial-immune interaction (Figure 1b). Bright-field microscopy examination confirmed the clear cell border of the differentiated colon monolayer after three days in GuMI (Figure 1c). The morphological inspection confirmed the presence of dendritic cells and macrophages in GuMI-APC (Figure 1d–f) but not in GuMI without APCs(Figure 1c). These results suggest that dendritic cells and macrophages successfully adhered to the bottom of the porous membrane and to the colonic epithelium. Notably, the TEER values of epithelium with and without APCs were not significantly different after three days of co-culture (Figure 1g), indicating that APCs do not alter the barrier function.

### Antigen-presenting cells are essential components of an immune-competent *in vitro* mesofluidic GuMI system

**Figure 2.**
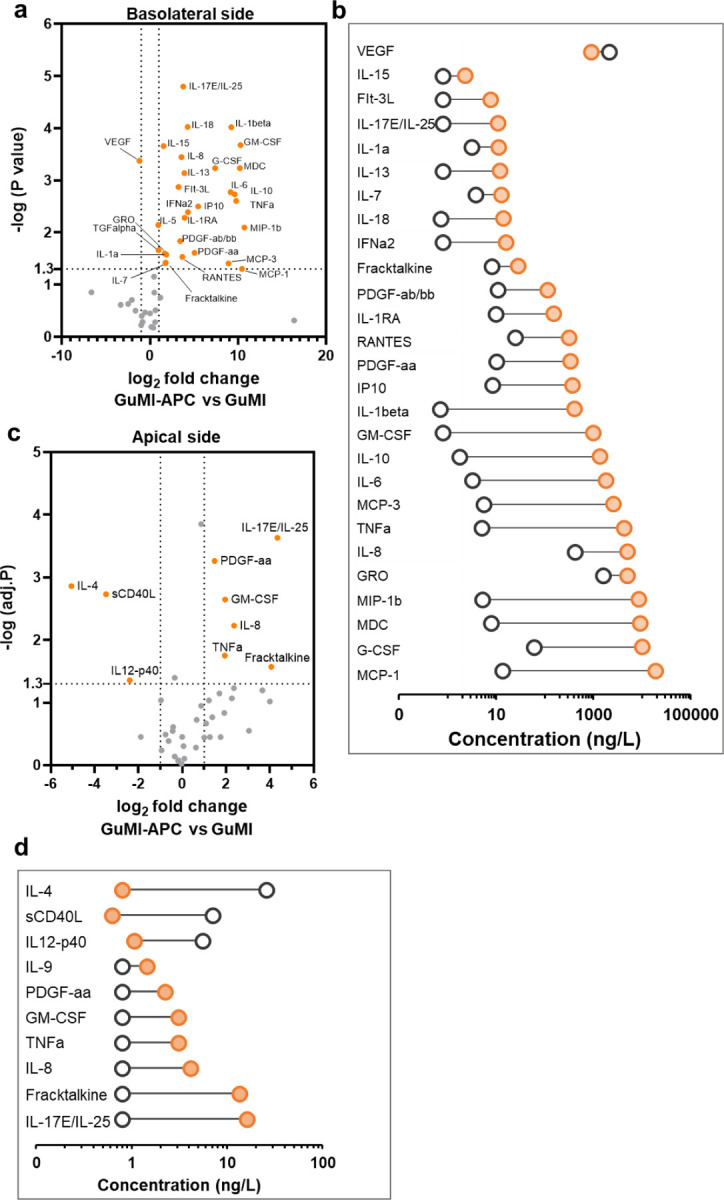
Antigen-presenting cells restore baseline cytokines in both apical and basolateral sides in the GuMI system. (**a**) volcano plot analysis for the cytokine profile in the presence versus the absence of APCs (i.e., dendritic cells and macrophages) in the apical compartment of GuMI. Orange-filled circles indicate the significantly changed cytokines. (**b**) list of significantly increased cytokines and decreased VEGF in the apical compartment of GuMI-APC (orange-filled circle) versus GuMI (black hollow circle). (**c**) volcano plot of cytokine profile in the basolateral compartment of GuMI-APC versus GuMI. Orange-filled circles indicate the significantly changed cytokines. (**d**) list of significantly increased and decreased cytokines in the basolateral compartment of GuMI-APC (orange filled circle) versus GuMI (black hollow circle). n = 2–3.

Next, we asked if the presence of immune cells contributes to the baseline immune responses. We compared the secreted cytokine and chemokine profiles in the presence and absence of APCs in GuMI (Figure 2). We determined the cytokine concentration in both apical and basolateral media collected 72 h after in GuMI-APC and GuMI. Because no lingua franca is accepted in the nomenclature of macrophage activation and polarization,^24^ we did not classify the macrophages or dendritic cells into specific activation categories such as M1 or M2. Instead, we reported the changes in the secreted cytokines. For ease of discussion, we keep the widely used original and systematic names of genes or proteins, for example, MCP-1/CCL2, in the discussion below.

In the basolateral side where APCs reside, cytokines were found to be changed to a great extent in the presence of APCs. Of the 47 analyzed cytokines/chemokines, 28 were significantly increased (adj. p<0.05, │log2fold change│ ≥1), and one (VEGF) was significantly decreased in GuMI-APC versus GuMI (Figure 2a). These analytes include hallmark cytokines secreted by dendritic cells and macrophages, i.e., MCP-1/CCL2, MIP-1a/CCL3, MCP-3/CCL7, G-CSF/CSF3, IL-6, IL-10, GM-CSF/CSF2, PDGF-aa, RANTES/CCL5, IL-18, IL-13, FGF2, IL-17E/IL-25. Notably, MCP-1/CCL2 on the basolateral side increased from 13 ng/L to 18815 ng/L (Figure 2b), suggesting APCs are the primary source of MCP-1/CCL2. These results are consistent with previous observations that macrophages isolated from mouse intestinal lamina propria produce MCP-1/CCL2 even without inflammation,^25^ and intestinal epithelial cells also produce MCP-1/CCL2.^26^ Macrophages also play an essential role in intestinal homeostasis by producing anti-inflammatory cytokine IL-10. Herein, we employed M-CSF-induced PBMC-derived macrophages. In GuMI-APC, a high amount (1385 ng/L) of IL-10 but not IL-12 and IL-23 (Figure 2a and 2b) was observed on the basolateral side. Similarly, Kamada et al. observed that M-CSF-induced bone marrow-derived macrophages and colonic lamina propria macrophages produced a high amount (~1500 ng/L) of IL-10 but not IL-12 and IL-23 upon stimulation of heat-killed bacteria *Enterococcus faecalis*, whereas GM-CSF-induced counterparts secreted a large amount of IL-12 and IL-23.^27^

Dendritic cells also contribute to producing specific cytokines IL-6, IL-18, TNF-α, and GM-CSF/CSF2 (Figure 2a and 2b). It was shown that dendritic cells can spontaneously expressed IL-1α, IL-1β, IL-6, IL-7, IL-12 (p35 and p40), IL-15, IL-18, TNF-α, TGF-β, M-CSF/CSF1, and GM-CSF/CSF2, but not IL-2, IL-3, IL-4, IL-5, IL-9, and IFN-γ transcripts.^28^ Consistently, we did not observe significant increase of IL-2, IL-3, IL-4, IL-5, IL-9, and IFN-γ protein in the basolateral side of GuMI-APC. Importantly, both anti- and pro-inflammatory cytokines IL-10, IL-8, and TNF-α were increased, likely due to the baseline immune responses of APCs. Both anti- and pro-inflammatory cytokines are maintained at a certain level in homeostasis *in vivo* in the colon.^25^ This and the macrophage-derived cytokines suggest that macrophages and dendritic cells function under the oxygen gradient and fluidic microenvironment. It also demonstrates that APCs largely contribute to secreted growth factors, cytokines, and chemokines, highlighting the importance of APCs in establishing an immune-competent *in vitro* gut model.

In contrast to the basolateral side, we did not expect the detection of cytokines or chemokines in the apical side because the media was refreshed at a flow rate of 10 µl/min, equivalent to a ~123 fold dilution over two days. Surprisingly, integrating APCs in GuMI significantly increased the number and levels of detectable cytokines on the apical side (Figure 2c), suggesting that APCs are metabolically active and have baseline immune responses. Interestingly, three cytokines, i.e., sCD40L, IL12-p40, and IL-4 (Figure 2d), were significantly decreased on the apical side but not changed on the basolateral side of GuMI-APC. IL-4 effectively promotes the differentiation of dendritic cells and is known to be consumed during the activation of dendritic cells.^29^ Following IL-4-mediated differentiation, dendritic cells can be driven to a more mature state by TNF-α.^30^ Consistently, TNF-α is significantly increased on the apical side (Figure 2b). Previous studies have reported that sCD40L, IL12-p40, and IL-4 are essential for differentiating monocytes toward dendritic cells and macrophages. Together with our data, these results suggest that dendritic cells and macrophages consumed sCD4L, IL12-p40, and IL-4. Notably, the APCs in the system are functionally secreting characteristic cytokines upon consuming the others. For instance, seven cytokines were significantly increased (Figure 2b), including PDGF-aa, GM-CSF/CSF2, TNF, IL-8/CXCL8, fractalkine/CX3CL1, and IL17E/IL-25. These cytokines are characteristic markers of functioning APCs reported in previous studies. MIP-1a/CCL3 and MIP-1b belong to the MIP-1 CC chemokine subfamily and were shown to be mainly secreted by dendritic cells and macrophages.^31^ TNF-α can increase secreted IL-8/CXCL8 in the basolateral side.^32^ IL-8/CXCL8 is secreted and is an essential mediator of innate immune responses. In mice, colonic lamina propia macrophages produce a large amount of IL-10 and MCP-1/CCL2 in a steady state and an even higher level of MCP-1/CCL2 in the inflammation site.^25^

### *F. prausnitzii* induces transcriptional immune responses in the colonic epithelium in the presence of APC

**Figure 3.**
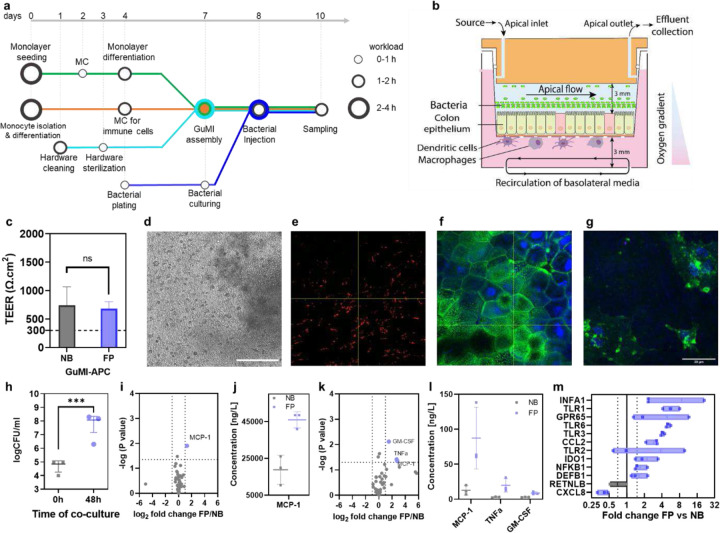
Cytokine and transcriptional changes induced by bacterium *F. prausnitzii* in GuMI with APCs. (**a**) workflow of GuMI experiments including preparation of monolayer (green line), monocyte isolation and APC differentiation (orange line), hardware preparation (aqua), and bacterial culturing (blue line). Circles in the metro map indicate the critical tasks and the workload. (b) schematic demonstration of co-culture of *F. prausnitzii*, colonic epithelium, and APCs. (c) TEER values of GUMI-APC with and without co-culture of *F. prausnitzii*. (d-g) brightfield image (d), immunofluorescent staining of bacterial cells (e), epithelium (f), and APCs (g). (h) live bacterium *F. prausnitzii* density at 0 and 48 hours. (i) volcano plot comparing cytokines/chemokines in apical media in the presence and absence of *F. prausnitzii* (GuMI-APC-FP vs. GuMI-APC-NB). (**j**) the significantly increased cytokine concentration in apical media shown in (i). (**k**) volcano plot on the comparison of cytokines or growth factors in basolateral media in the presence and absence of *F. prausnitzii* (GuMI-APC-FP vs. GuMI-APC-NB). (**l**) the concentration of significantly increased cytokine MCP-1/CCL2 in basolateral media shown in (k). (**m**) transcriptional change of inflammation-related genes in colonic epithelial cells in GuMI-APC *F. prausnitzii* versus no bacteria. n = 3. Blue boxes indicate a <0.75-fold decrease, and black boxes indicate no significant difference.

Next, we ask if this APC-epithelium co-culture could accommodate oxygen-intolerant anaerobe *F. prausnitzii*. *F. prausnitzii* is one of the most oxygen-sensitive bacterial species in the human adult gut microbiota. We introduced the bacterium 16–18 hours after priming the whole system (Figure 3a and 3b). The co-culture of *F. prausnitzii* in GuMI-APC requires careful coordination and synchronization of different tasks (Figure 3a). To unambiguously determine if *F. prausnitzii* grows in the co-culture with colonic epithelium and APCs, we determined the concentration of live bacterial cells by counting the colony-forming unit (see Method). The intact monolayer can prevent oxygen from leaking from the basolateral to the apical side. The intactness of the barrier function is verified by TEER measurement and visual inspection under a microscope. No significant change was observed in the TEER values (Figure 3c), and no observable holes were observed under microscopic examination (Figure 3d–g), confirming that the monolayers were intact with or without APCs. This intact epithelial barrier can support the growth of *F. prausnitzii* after 48 hours of co-culture in GuMI-APC. Upon introduction in GuMI-APC, the concentration of *F. prausnitzii* is ~10^5^ CFU/ml, which increased to ~10^8^ CFU/ml after 48 hours of co-culture (Figure 3h). This result indicates an active bacterial growth in GuMI-APC. The bacterial concentration is similar to the density observed in GuMI without APCs.^33^. To further investigate the spatial organization of the different cells in GuMI-APC, we performed immunofluorescent staining for the cells in the co-culture. Strikingly, many bacteria cells line the top of the colonic epithelium (Figure 3e). Beneath the bacterial cell “layer”, the monolayer displayed a cobblestone arrangement of the colonic epithelium (Figure 3f), similar to the colonic epithelium in other reports.^10,33–35^ APCs, such as dendritic cells with irregular shapes and tentacle-like extensions (Figure 3g), were found beneath the membrane. Colony plating indicated a more than 1000-fold increase in the density of bacteria in the apical chamber of GUMI-APC, which reached ~10^8^ CFU/ml (Figure 3h). This final bacterial density is similar to that in the absence of APCs,^33^ suggesting that the introduction of APCs does not influence the growth of *F. prausnitzii*. These results suggest a successful co-culture of bacteria-epithelium-APCs leading to an immune-competent GuMI platform.

Next, we sought to investigate the impact of *F. prausnitzii* on the immune responses of colonic epithelium in the presence of APCs. We compared the concentration of 47 cytokines in the apical and basolateral compartments of GuMI-APC-FP vs. GuMI-APC-NB (no bacteria, Figure 3). Surprisingly, most cytokines remained similar to the baseline levels in GuMI-APC-NB (Figure 3i–j). Only three cytokines were significantly increased in the apical and one in the basolateral compartments of GuMI-APC-FP, respectively (Figure 3i and 3k). The protein levels of MCP-1/CCL2 in both apical and basolateral media were significantly increased by *F. prausnitzii* (Figure 3j and 3l). Lactobacilli and streptococci induce MCP-1/CCL2 production in human macrophages.^36^ MCP-1/CCL2 is critical in recruiting monocytes in the inflammation site.^37^ MCP-1/CCL2 protein is constitutively secreted in the normal intestinal colonic mucosa and is up-regulated in patients with Ulcerative Colitis or Crohn’s Disease.^26^ Consistently, the mRNA level of MCP-1/CCL2, 2.9-fold) was significantly increased in colonic epithelial cells in GuMI-APC-FP (Figure 3m). This result agrees with the clinical observations, where MCP-1/CCL2 mRNA levels were markedly increased in inflamed intestinal biopsies from patients with inflammatory bowel disease. ^26^ In addition to MCP-1/CCL2, TNF-α protein was also increased in GuMI-APC-FP (Figure 3l). Recently, it was found that 10% of *F. prausnitzii* fermented supernatant increased the protein level of TNF-α in LPS-pretreated colonic HT29 cells.^38^ These results indicate that *F. prausnitzii* homeostatically actives immune and epithelial cells in the GuMI-APC, with increased secretion of a few pro-inflammatory cytokines. To test this hypothesis, we looked at the expression of genes that are critical for inflammation regulation such as TLRs, IDO1, IFNA1, CXCL8/IL8, and NFKB1 in colonic epithelium. TLRs mediate host cell recognition of virus, pathogens, and commensal bacteria,^39^ with genes such as IDO1 and IFNA1 regulating TLR expression.^40,41^ Transcription of IDO1 is significantly higher in the ileum and colon in models of inflammation induced by immunostimulatory DNA (CpG), TNBS, and DSS.^42^ Mice deficient in IDO1 repress the activation of the TLR-Myd88-NFKB1 network and thus developed less severe colitis induced by DSS.^41^ Herein, we found that the transcription of TLR1, TLR3, TLR6, and NFKB1 was significantly upregulated in colonic epithelial cells upon exposure to *F. prausnitzii* (Figure 3m). Consistently, the transcription of IDO1 and IFNA1 was increased by *F. prausnitzii*. The activation of IDO1 in colonic epithelial cells agrees with previous observations on *F. prausnitzii*-mediated activation of dendritic cells.^43^ When dendritic cells were exposed to a single dose of dead *F. prausnitzii* cells, IDO1 and TLRs (i.e., TLR2 and TLR4) were activated at the transcriptional level.^43^ Despite *F. prausnitzii* unexpectedly decreased the mRNA level of CXCL8/IL8 (0.4-fold, Figure 3m) with no change in CXCL8/IL-8 secretion, these results indicate that in the presence of APCs, *F. prausnitzii* primes colonic epithelial cells to be transcriptionally activate toward bacteria-activating and pro-inflammatory states, but to secrete only a few pro-inflammatory cytokine proteins.

### CD4^+^ naïve T cells increase the *F. prausnitzii*-induced secretion of cytokines and decrease the transcription of TLR in the colonic epithelium

**Figure 4.**
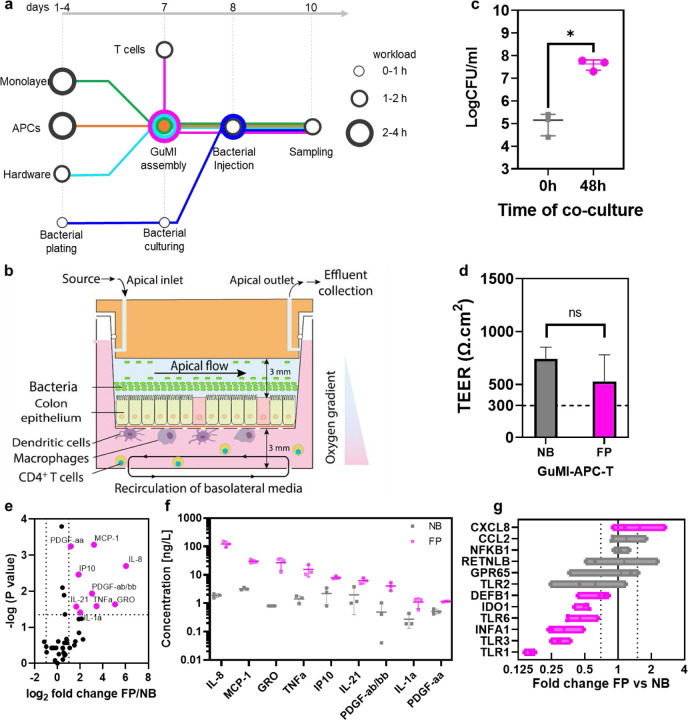
Successful integration of CD4^+^ naïve T cells in GuMI-APC demonstrates the contribution of CD4^+^ naïve T cells in the systemic immune response to bacterium *F. prausnitzii*. (a) workflow to establish GuMI-APCT with and without *F. prausnitzii*. (b) illustration of designed co-culture of colonic epithelium, APCs, CD4^+^ T cells, and *F. prausnitzii* in the GuMI platform. (c) the introduction of CD4^+^ T cells does not affect the live bacterial density of *F. prausnitzii* in the apical compartment. CFU: colony forming unit. (d) The introduction of CD4^+^ T cells does not influence the TEER values of the monolayer in GuMI. TEER: transepithelial electrical resistance. (**e**) The volcano plot compares cytokines/chemokines in apical media in the presence and absence of *F. prausnitzii* (GuMI-APCT-FP vs. GuMI-APCT-NB). (**f**) significantly increased cytokines in apical media induced by *F. prausnitzii* in GuMI-APCT. (**g**) transcriptional change of selected inflammation-related genes induced by *F. prausnitzii* in GuMI-APCT.

In the intestinal mucosal barrier, the interaction of APCs and T cells is crucial in the intestinal innate immunity in response to microbiota. A recent single-cell survey revealed that T cells (including CD4^+^ T cells, Th1 helper cells, Th17 cells, and other Treg subtypes) account for a considerable proportion of the immune cell population in human colon mucosa.^44^ Reasoning that adding T cells will likely close the communication gap among the epithelium, APCs, and T cells responding to the gut microbiota, we integrated CD4^+^ naïve T cells into the established GuMI-APC to generate GuMI-APCT co-culture and exposed the system to *F. prausnitzii* (GuMI-APCT-NB *vs.* GuMI-APCT-FP, Figure 4a). The pumping system allows the recirculation of CD4^+^ T cells in the basolateral compartment without causing cell death or damage (Figure 4b),^10,45^ and the designed co-culture enables the interactions of colonic epithelial cells, APCs, CD4^+^ T cells, and bacterial cells (Figure 4b). As a quality control, we first examined if *F. prausnitzii* grows similarly to that in GuMI-APC. At 48 h after bacterial introduction, the live bacterial density in the apical compartment reached ~10^8^ CFU/ml in GuMI-APCT-FP, which is similar to that in GuMI-APC-FP (Figure 4c) and that of FP in human fecal samples (2.5 × 10^7^ −7.9 × 10^11^ gene copies/g feces) ^46^ and mouse intestine (3.4 × 10^8^ to 2 × 10^9^ CFU/g).^47^ Consistently, the integrity of the colonic epithelial monolayer was not changed by CD4^+^ naïve T cells, evidenced by the similar TEER values above 300 Ω cm^2^ (Figure 4d), an empirical threshold for an intact epithelial barrier *in vitro*.^35,48^ These results confirm that naïve CD4^+^ T cells do not affect bacterial growth nor epithelial barrier integrity.

Next, we sought to investigate how the GuMI-APCT responds to *F. prausnitzii*. We first looked at the changes in cytokines induced by *F. prausnitzii*. Nine of 47 cytokines/chemokines in the apical compartment were significantly increased by *F. prausnitzii*, while no cytokines/chemokines were significantly decreased (Figure 4e). TNF-α and MCP-1/CCL2 were increased by *F. prausnitzii* in GuMI-APCT, similar to that in GuMI-APC. With the presence of CD4^+^ naïve T cells, six other cytokines, i.e., IL-8, GRO, IP10, IL-21, PDGF-ab/bb, IL-1A, and PDGF-aa, were increased in response to *F. prausnitzii*. The cytokines induced by *F. prausnitzii* are typically regarded as pro-inflammatory cytokines. However, the levels of these cytokines are way below the levels considered to be hyper-inflammation. In fact, it is believed that commensal gut microbiota contributes to the training of our immune system by inducing baseline inflammation during homeostasis. Nevertheless, including the CD4^+^ T cells enhances the cytokine-mediated immune responses to *F. prausnitzii*, suggesting active communication among APCs, T cells, and epithelial cells. Transcriptional changes in epithelial cells further support this notion. In the presence of naïve CD4^+^ T cells, *F. prausnitzii* downregulated the transcription of IDO1 (0.49-fold) and IFNA1 (0.33-fold) in colonic epithelial cells. Similarly, TLRs, the downstream genes of IDO1 and IFNA1, were decreased: TLR1 (0.16-fold), TLR2 (0.63-fold), TLR3 (0.30-fold), and TLR6 (0.45-fold). Importantly, no dramatic change were observed for NFKB1 (1.1-fold). These results suggest that *F. prausnitzii* is lowering the inflammation state at the transcriptional level in the presence of naïve CD4^+^ T cells. Compared to the circumstance when only APCs were present, the addition of naïve CD4^+^ T cells reverses the *F. prausnitzii*-mediated effects in colonic epithelium at the transcriptional level, highlighting the importance of T cells in coordinating the innate immune response to *F. prausnitzii* in the GuMI system. Interestingly, IDO1 is significantly higher in ileal tissue from Crohn’s Disease patients with active inflammation but not without active inflammation.^42^ Transcription of IDO1 is significantly higher in the ileum and colon in rodent models of inflammation induced by immunostimulatory DNA (CpG), TNBS, and DSS.^42^ It has been shown previously that TLR3 and TLR4 were significantly downregulated by *F. prausnitzii*-produced butyrate during its co-culture with colonic epithelium in the same GuMI physiomimetic platform.^35^ *F. prausnitzii* can produce several types of anti-inflammatory molecules such as butyrate, MAM,^49^ and sialic acid.^47^ In animal models, *F. prausnitzii* alleviated the IBD symptoms.^50^ Our results agree with these observations and demonstrate the contribution of naïve CD4^+^ T cells in the interaction of the epithelium, innate immune components, and *F. prausnitzii*.

In summary, we established a mesofluidic microphysiological system that enables the co-culture of primary human colonic epithelium with monocyte-differentiated antigen-presenting cells, CD4^+^ naïve T cells, and oxygen-sensitive gut commensal *F. prausnitzii*. Using multiplex cytokine assays and RT-qPCR, we demonstrated that the antigen-presenting cells substantially contribute to maintaining systemic immune cytokines. On top of that, circulating CD4^+^ naïve T cells alter systemic cytokine-mediated immune responses to *Faecalibacterium prausnitzii* and transcription of microbe-recognition genes. The results demonstrate the successful integration of three types of immune cells that are important in the innate immune-epithelium-microbiota axis and reveal the contribution of individual types of immune cells in response to gut commensal *F. prausnitzii*. These findings elucidate the critical role of CD4^+^ T cells that may maintain tolerance to intestinal microbiota by rendering the sensitivity of APCs and intestinal epithelial cells to commensal bacteria through the downregulation of proinflammatory genes. Finally, the established system provides a new tool to study microbe-host-immune interactions in the context of health and disease.

## Materials and Methods

### Bacterial culture

*Faecalibacterium prausnitzii* A2-165 (also known as *Faecalibacterium duncaniae* DSM17677) was obtained from the Harvard Digestive Disease Center. The strain's identity was confirmed using Sanger sequencing (see below). Bacteria from glycerol stock were plated in yeast casitone fatty acid (YCFA) agar (Anaerobe Systems, AS-675) for 24–48 h after being cultured at 37 °C in the incubator inside the anaerobic chamber (Coy Laboratory), and a colony was picked and cultured in Hungate tubes containing liquid YCFA medium (Anaerobe Systems, AS-680). O_2_ in the anaerobic chamber was constantly removed by the Palladium Catalyst (Coy Laboratory, #6501050), which was renewed biweekly by incubating the catalyzer in the 90 °C oven for two days.

### PCR and Sanger sequencing

Bacteria identity was confirmed by Sanger sequencing by following the established protocol.^35^ Briefly, bacterial cells were pelleted by centrifugation (12000 g × 5min). The DNA was extracted using the GeneElute bacterial DNA kit (NA2110, Sigma-Aldrich) following the manufacturer's protocol. Afterward, PCR was performed in triplicate to amplify 16s rDNA using DreamTaq Green PCR Master Mix (K1081, Thermo Fisher Scientific Inc.) with primers F8 (5’-AGTTTGATCCTGGCTCAG-3’) and 1492R (5’-TACGGYTACCTTGT TACGACTT-3’) by following the procedures described elsewhere.^51^ PCR products were sent for Sanger sequencing after DNA purification (Genewiz Inc.). The identity of the bacteria was confirmed to be *F. prausnitzii* DSM17677 using Blastp (Figure S1).

### Colonic epithelial monolayer

#### Colon Organoid and Monolayer Culture

Primary human colon organoids and monolayers were established and cultured according to previously described protocols.^35,52^ The organoids were derived from endoscopic tissue biopsies taken from a patient (the normal appearing region of rectosigmoid sample from a 30-year-old male patient for diverticulosis and diverticulitis) upon informed consent. Methods performed followed the Koch Institute Institutional Review Board Committee and the Massachusetts Institute of Technology Committee on using humans as experimental subjects. The medium for maintaining organoids and monolayers includes a base medium, organoid growth medium, seeding medium, and differentiation medium. The recipe for each medium is listed in Table S2. In brief, organoids in Matrigel (growth factor reduced, phenol red-free; Corning, 356231) droplets were grown in 24-well tissue culture-treated plates (Olympus Plastics, 25–107) and passaged every seven days at a 1:3 split ratio. A medium change was performed on day four using organoid growth medium after passaging. To prepare the monolayer, organoids were collected on day seven and pelleted by centrifugation (1000 g × 5min, 4°C), followed by Matrigel digestion using Cell Recovery Solution (Corning, 354253; 1mL per 100 µL Matrigel). The resulting organoid suspension was then incubated on ice for 45–60 min, pelleted, and then digested at 37 °C for 5 min using 1mL Trypsin/EDTA (2.5mg/mL Trypsin [Sigma, T4549] and 0.45 mM EDTA [Ambion, AM9260G] in PBS without calcium and magnesium [PBS^−/−^, Gibco, 10010-023]). The digested organoids were manually dissociated into single cells using a 1000-µL pipette with a bent tip. The resulting cell suspension was then pelleted (300 g × 5 min, 4°C) after neutralizing Trypsin was neutralized with 10% FBS in base medium. The cell pellet was resuspended in the seeding medium and seeded in collagen I-coated (Gibco, A10483-01, 50 µg mL^−1^ in PBS) 12-well Transwells. The seeding cell density was 300,000 cells per well (surface area: 1.12 cm^2^). Around 72 hours after seeding, the monolayers were differentiated by switching to the antibiotic-free base medium on the apical side and the differentiation medium on the basolateral side. After switching to differentiation medium, the monolayers were cultured for four days (a total of seven days), with medium change on day five. The monolayers were used for experiments on day seven after seeding (day four after differentiation).

### Generation of dendritic cells, macrophages, and CD4^+^ T cells

#### Isolation and differentiation of monocytes

Monocytes were isolated from peripheral blood mononuclear cells (PBMC). PBMC were isolated from fresh whole blood with CPDA-1 anticoagulant (Research Blood Components LLC) using the SepMate PBMC Isolation Kit (StemCell, 85450) following the manufacturer protocol. After isolation, the PBMC were suspended in an immune cell freezing medium (RMPI with 10% dimethyl sulfoxide (DMSO) and 10% heat-inactivated FBS) and frozen at −196 °C.

Monocytes were isolated and differentiated into dendritic cells and macrophages on the 7th day before device assembly (see details below) for seven days. First, the PBMC was thawed in a 37° C water bath for approximately 1 minute before diluting 1:10 in PBS^−/−^ containing 2% heat-inactivated FBS (PBS-HIFBS). After that, cells were pelleted (300 g × 5 min, 4°C) and then resuspended in 1 mL of PBS-HIFBS, followed by a transfer into a 5-ml round-bottom polystyrene tube (StemCell, 100-0088). An additional 1.5 mL PBS-HIFBS was used to recover the residual cells and transferred into the same polystyrene tube. The isolation of monocytes was performed using the EasySep^™^ Human Monocyte Enrichment Kit without CD16 depletion (StemCell, 19058) and EasySep^™^ Magnet (StemCell, 18000). The resulting monocytes were split into two aliquots and pelleted (300 g × 5 min, 4°C). The cell pellets were resuspended in the macrophage or dendritic cell differentiation media and cultured in 24-well tissue-treated plates. Both dendritic cells and macrophages were plated in 24-well, tissue culture-treated plates at a density of 1*10^6^ cells per well and in a volume of 500 μl per well. Four days after isolation and plating, 500 μl of MDM and DCDM were added to each macrophage and dendritic cell well, respectively. Dendritic cells were mixed gently via repeated pipetting upon media change to disrupt cell clumps, while macrophages were not mixed. 25,000 of each cell type were used to attach the membrane.

#### Isolation of CD4^+^ naïve T-cells

The CD4^+^ naïve T cells were isolated from PBMC on the same day of device assembly (see details below). In brief, PBMCs were thawed in a 37° C water bath for approximately 1 minute before diluting 1:10 in PBS-HIFBS. After that dilution in the isolation buffer, centrifugation at 300 g for 5 min and 4°C was performed, and the isolation buffer was removed from the cell pellet. The cell pellet was then resuspended in 1 mL of PBS-HIFBS and transferred into a 5-ml round-bottom polystyrene tube (StemCell, 100-0088). An additional 1.5 mL PBS-HIFBS was used to recover the residual cells and transferred into the same polystyrene tube. Naïve CD4+ T cells were isolated using the EasySep^™^ Human Naïve CD4^+^ T Cell Isolation Kit II (StemCell, 17555) and EasySep^™^ Magnet (StemCell, 18000). Once isolated, the naïve CD4^+^ T cells were pelleted and resuspended in 1 mL of RPMI 1640 supplemented with 10% HIFBS (RPMI-HIFBS) and ready for use. Cells were counted via Trypan Blue and Countess II Automated Cell Counter, and 60,000 naïve CD4^+^ T cells were used in each well in circulation.

#### Co-culture of epithelial monolayers with dendritic cells, macrophages, and naïve CD4^+^ T cells

In the experiments with APCs, i.e., dendritic cells and macrophages were harvested and seeded onto the basolateral side of the transwell membrane. To harvest the cells, cells were resuspended in their own media and collected into a conical tube (one tube per cell type). The residual cells were detached by adding 250 μl TrypLE Express (Gibco, 1260413) to each well and incubated at 37° C for approximately 15 minutes, or until cells were detached from the plate. The TrypLE was then neutralized with 750 μl RPMI-HIFBS. The resulting cell suspensions were collected into the corresponding conical tubes. After that, the cells were pelleted (300 g × 5 min, 4°C), resuspended in 1 mL of RPMI-HIFBS, and counted using trypan blue and countess. Dendritic cells and macrophages were then combined to achieve a density of 1.67*10^5^ cells per mL for each cell type. Before adding dendritic cells and macrophages, the media of the transwells was removed from both apical and basolateral sides, and each side was rinsed once with an antibiotic-free base medium. The transwells were then inverted and placed in a petri dish before adding 150 μl of the dendritic cell and macrophage cell suspension to each well to achieve a density of 0.25*10^5^ cells per transwell for each cell type. The transwells were then incubated at 37° C for 2 hours to allow the attachment of dendritic cells and macrophages before proceeding with the further experimental setup.

In the experiments with naïve CD4^+^ T cells, the freshly isolated naïve CD4^+^ T cells were pelleted and resuspended using colon differentiation medium to achieve a density of 40,000 cells/mL (60,000 cells per well). The T-cell-containing medium was added into the basolateral compartment in the GuMI device, where the naïve CD4^+^ T cells were circulated.

### Device assembly and operation

The device assembly, operation, and sampling followed the previously described protocol^33^ with slight adaption in the experiments with immune cells. In brief, all components of the GuMI device were sterilized by autoclave (121 °C, 45 min), except the pneumatic plates, which were sterilized with ethylene oxide. Then, the device was assembled under sterile conditions. The GuMI apical medium (110 mL 10% YFCA in PBS^+/+^) was added to the apical source reservoir on top of the GuMI device (total capacity 150 mL). The medium in the apical source reservoir was then deoxygenized with 5% CO_2_ and 95% N_2_ for 45–60 min before being introduced into the apical inlet. After that, the apical inlet of the Transwell was temporally blocked with a 200-µl pipette tip to force the deoxygenized apical medium to flow out of the injection port, which was then sealed with an injection septum and a customized stainless-steel hollow screw. The pipette tips were then removed. The colonic epithelial monolayers were transferred to the six basolateral reservoirs prefilled with PBS^+/+^. The base medium in the apical side of the monolayers was replaced with the 10% diluted YCFA in PBS^+/+^. Then, the entire basal plate was integrated with the apical plate using the lever. In the experiment with APCs or APCs and naïve CD4^+^ T cells, the inverted transwells were reversed and placed into basolateral reservoirs prefilled with PBS^+/+^. The basal plate was then disassembled using the lever, and the PBS was replaced with colon differentiation media, or CD4^+^ T cells colon differentiation media in the experiments with CD4^+^ T cells. The system was primed for 24 h in a cell culture incubator while the medium in the apical source reservoir was constantly purged with 5% CO_2_ and 95% N_2_. The recirculation flow rate in the basal compartment was 5 µl/min, and the apical flow rate was 10 µl/min. The effluent was cleared every 24 h with a 10-ml syringe (302995, BD Biosciences) throughout the experiments.

### Bacteria co-culture with colonic epithelial monolayers

The co-culture of bacterial cells with colonic epithelial monolayer was performed according to the established protocol.^52^ Briefly, colonic epithelial monolayers were cultured in the GuMI device for 24 h before adding bacteria. In the experiments with APCs, the monolayers were replaced by the monolayers with dendritic cells and macrophages attached to the bottom of the porous polyester membrane. After that, the overnight grown bacterial cultures were diluted 1000 times with a pre-reduced YCFA medium. Approximately 1 ml of the diluted bacterial cells was slowly injected into the apical channel. After one hour of settling the bacterial cells, the flow resumed on both the apical and basolateral sides. After the experiment, the whole device was transferred to a biosafety cabinet, and the basal plate was carefully disassembled. The sealed Transwells were individually removed from the apical plate and placed onto a new 12-well plate. Immediately after that, the apical medium was collected using a 1-ml syringe with a short needle (305122, BD Biosciences) and then immediately injected into a 20-ml pre-reduced and autoclaved HDSP vial (C4020-201, Thermo Scientific) sealed with 20-mm Crimp Cap (95025-01-1S, MicroSolv). All the vials were transferred into an anaerobic chamber, where 10 µl of the apical medium was used for CFU counting on agar plates. The rest of the medium was transferred into a 1.5-ml polypropylene tube, where bacterial cells were pelleted in a microcentrifuge (14000 g × 5 min). The supernatant was transferred into a new 1.5-ml tube. All samples were stored at −80 °C until further analysis.

The Transwells were washed twice with PBS+/+ (14040182, Thermo Scientific) in both apical and basolateral sides to completely remove the cell-culture medium and the residual bacterial cells before bright field imaging and TEER measurement. After aspirating the PBS^+/+^, 350 μl of 1% 2-mercaptoethanol solution was added to the apical side, followed by incubation for 10 min at room temperature. One volume of 70% ethanol was then added and mixed homogeneously, and the mixture was collected and stored at −80 °C until further analysis.

### Multiplex cytokine/chemokine assays

The concentration of autocrine factors, cytokines, and chemokines in the apical media was measured using customized MULTIPLEX MAP assays, 47-plex human cytokine/TH17 panel (EMD Millipore) adapted from the previous protocol. Briefly, samples were measured at multiple dilutions to ensure the measurements were within the assay's linear dynamic range. We reconstituted the protein standard in the same media and serially diluted the protein stock to generate a 7-point standard curve. Assays were run on a Bio-Plex 3D Suspension Array System (Bio-Rad Laboratories, Inc.). Data were collected using the xPONENT for FLEXMAP 3D software, version 4.2 (Luminex Corporation, Austin, TX, USA). The concentration of each analyte was determined from a standard curve that was generated by fitting a 5-parameter logistic regression of mean fluorescence on known concentrations of each analyte (Bio-Plex Manager software).

#### RNA extraction and Reverse transcription-quantitative polymerase chain reaction (RT-qPCR)

Prior to RNA extraction, the cell lysate in 1% 2-mercaptoethanol solution was mixed with one volume of 350 µl of 70% ethanol and pipetted to a homogeneous mixture. Then, total RNA was extracted using a PureLink RNA mini kit (ThermoFisher, 12183020) by following the manufacture protocol, except treating samples with PureLink DNase (ThermoFisher, 12185010) during one of the wash steps to remove DNA.

RT-qPCR was performed to quantify gene expression. Briefly, the mRNA was converted to cDNA using the High-Capacity RNA-to-cDNA Kit (Thermo Fisher Scientific, 4387406). TaqMan Fast Advanced Master Mix (Thermo Fisher Scientific, 4444557) and TaqMan probe were mixed in MicroAmp EnduraPlate Optical 96-well fast clear reaction plate with barcode (Thermo Fisher Scientific, 4483485) according to manufacture protocol. TaqMan probes used in this study are available in Table S3.

#### Immunofluorescence staining

The immunofluorescent staining of the monolayers was carried out based on the procedures described previously.^33^ Briefly, monolayers taken off the platform were immediately fixed with 4% formaldehyde for 10 minutes following a very gentle sampling of the apical medium. The samples were then permeabilized with 0.2% Triton-X for 10 minutes. After permeabilization, the wells were washed once in PBS^+/+^ and immediately stained overnight with Phalloidin-iFluor 488 Reagent (ab176753-300TEST) and DAPI (1:1000) in Blockaid at 4 °C. After washing the samples with PBS^+/+^ for two times, the monolayers were excised and mounted on a coverslip using ProLong Gold antifade reagent (Thermo Fisher). Mounted samples were imaged with a Zeiss LSM800 confocal microscope.

#### Transepithelial electrical resistance measurement

EndOhm-12 chamber with an EVOM2 meter (World Precision Instruments) was used to measure the transepithelial electrical resistance (TEER) values.

## Supplementary Material

Supplement 1

## Highlights

- An immune-competent human gut-microbe-immune (GuMI) microphysiological system is established
- GuMI enables the co-culture of primary human colonic epithelium, antigen-presenting cells, CD4^+^ naïve T cells, and oxygen-intolerant bacterium *Faecalibacterium prausnitzii*
- Dendritic cells and macrophages are essential to secreted cytokines and chemokines
- The presence of CD4^+^ T cells alters immune responses to *Faecalibacterium prausnitzii* at cytokine and transcriptional levels
