# Supplementary material for: An immune-competent human gut microphysiological system enables inflammation-modulation of Faecalibacterium prausnitzii
Source: Res Sq. 2023 Oct 12:rs.3.rs-3373576. Preprint. [Version 1] doi: 10.21203/rs.3.rs-3373576/v1 (PMC10602192; doi:10.21203/rs.3.rs-3373576/v1)
Supplement: Supplement 1 [file NIHPPrs3373576v1-supplement-1.pdf]

Supplementary Files

This is a list of supplementary files associated with this preprint. Click to download.

- ZhangImmuneGuMIsupplementary.docx
